# Iron Metabolism and Brain Development in Premature Infants

**DOI:** 10.3389/fphys.2019.00463

**Published:** 2019-04-25

**Authors:** Yafeng Wang, Yanan Wu, Tao Li, Xiaoyang Wang, Changlian Zhu

**Affiliations:** ^1^ Department of Neonatology (NICU), Children’s Hospital Affiliated Zhengzhou University, Zhengzhou, China; ^2^ Henan Key Laboratory of Child Brain Injury, Institute of Neuroscience and Third Affiliated Hospital of Zhengzhou University, Zhengzhou, China; ^3^ Department of Clinical Neuroscience, Center for Brain Repair and Rehabilitation, Institute of Neuroscience and Physiology, University of Gothenburg, Gothenburg, Sweden; ^4^ Department of Physiology, Sahlgrenska Academy, Institute of Neuroscience and Physiology, University of Gothenburg, Gothenburg, Sweden

**Keywords:** brain development, brain injury, iron metabolism, iron homeostasis, preterm infants

## Abstract

Iron is important for a remarkable array of essential functions during brain development, and it needs to be provided in adequate amounts, especially to preterm infants. In this review article, we provide an overview of iron metabolism and homeostasis at the cellular level, as well as its regulation at the mRNA translation level, and we emphasize the importance of iron for brain development in fetal and early life in preterm infants. We also review the risk factors for disrupted iron metabolism that lead to high risk of developing iron deficiency and subsequent adverse effects on neurodevelopment in preterm infants. At the other extreme, iron overload, which is usually caused by excess iron supplementation in iron-replete preterm infants, might negatively impact brain development or even induce brain injury. Maintaining the balance of iron during the fetal and neonatal periods is important, and thus iron status should be monitored routinely and evaluated thoroughly during the neonatal period or before discharge of preterm infants so that iron supplementation can be individualized.

## Introduction

Iron is a transition metal with the ability to transport oxygen and transfer electrons, and it acts as a catalyst in the active sites of oxidases, oxygenases, and certain antioxidants. All cells require iron due to iron’s role in important physiological processes such as oxidative phosphorylation and energy metabolism. For example, the cytochromes and succinate dehydrogenase that play critical roles in the tricarboxylic acid cycle are iron-containing proteins (Bartnikas, 2012). The huge demand for iron in the late fetal and early postnatal period is for hemoglobin (Hb) synthesis; however, iron’s function should not be undervalued during the development of all other organ systems. As with all nutrients, there is a greater requirement for iron during rapid growth and development.

Premature infants are at high risk of iron deficiency (ID) due to inadequate iron storage caused by the factors of preterm birth, early onset of postnatal erythropoiesis, and rapid growth after birth (Choudhury et al., 2015; Pettei et al., 2016). The lack of a gold standard to describe iron status clinically for healthy preterm infants is still a weakness. Previous studies showed lower iron storage in premature neonates compared with full term neonates, and the smaller premature neonates are at birth, the more susceptible they are to ID due to their proportionately smaller iron storage at birth (Haga, 1980; Schiza et al., 2007; Takala et al., 2010). Thus, most preterm infants need to be supplied with a certain dose of iron for the prevention of anemia of prematurity, and with iron supplementation and reasonable breast feeding, the situation of ID gradually improves with age (Takala et al., 2010; Schneider and Garcia-Rodenas, 2017). Cumulative evidence suggests that iron imbalance—both ID and iron overload—has negative consequences on infant development (Hare et al., 2015; Cusick et al., 2018). In this review article, we summarize the iron metabolism status in early life and its relation with brain development, and we focus especially on impact of iron dysregulation in preterm infants.

## Iron Metabolism and Regulation

Iron balance is strictly regulated by preventing both ID and iron overload. This homeostasis is achieved through iron storage, erythrocyte iron reutilization, and iron absorption (Finch and Huebers, 1982). Therefore, when the iron level of the body is inadequate, absorption is maximized, and when the iron level is adequate, iron absorption is restricted (Gkouvatsos et al., 2012). When iron is in overabundance, excess iron is kept in enterocytes as ferritin and in the liver, spleen, and bone marrow as hemosiderin (Saito, 2014). The ferroportin-mediated release of free iron ions into the plasma is essential for iron absorption, iron recycling, and overall iron homeostasis (Nemeth et al., 2004). Iron flux is controlled by hepcidin in the organs expressing ferroportin, and its expression is regulated by iron, hypoxia, inflammation, and other factors (Rivera et al., 2005; Chung et al., 2007; Vecchi et al., 2009). Conversely, ID, anemia, and hypoxia all inhibit hepcidin mRNA transcription (Vela, 2018), which results in unrestricted duodenal iron absorption and iron release from macrophages.

Under normal physiological conditions, protein-bound iron is the iron transport and storage form because it does not induce free radical reactions. However, protein-bound iron could be released from its binding proteins following perinatal asphyxia and/or postnatal hypoxia, and this is the common risk factor associated with brain injury in preterm infants (Albertsson et al., 2014; Laptook, 2016; Pillers, 2017). The blood pH decreases after asphyxia, causing transferrin to release iron and induce free radical production and iron accumulation, which could be seen in injured neurons and white matter (Rathnasamy et al., 2011; Beppu et al., 2014). These free radicals cause more iron to be released after mobilization from ferritin, and the reperfusion and reoxygenation after hypoxia could produce a great quantity of nitric oxide in the neonatal brain, causing the release of even more iron from its binding protein (Niatsetskaya et al., 2012). These mechanisms activate a cascade of iron release and free radical production that lead to extensive cellular oxidative stress and cell death (Shouman et al., 2008). After perinatal asphyxia and/or postnatal hypoxia, as an end product of lipid peroxidation, the serum levels of malondialdehyde are elevated in newborns (El Bana et al., 2016). The level of lipid peroxides and the severity of cell damage can be decreased by the iron chelator deferoxamine (Rathnasamy et al., 2011). It has also been suggested that iron-mediated ferroptosis might play an important role in preterm infants after perinatal asphyxia and/or postnatal hypoxia-induced brain injury (Wu et al., 2019).

## Iron Metabolism and Brain Development

**Figure 1:**
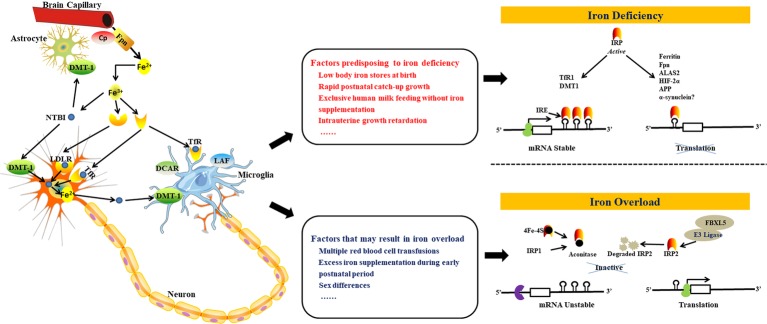
Schematic depiction of processes and pathways involved in iron homeostasis and regulation in the brain. Iron can enter the brain through the blood–brain barrier and the choroid plexus (1). Transport of iron across the blood–brain barrier is mediated by the TfR-DMT-1-Fpn pathway in a similar manner to cells in the periphery. Fe^2+^ released from the basolateral surface of brain capillary endothelial cells by Fpn is rapidly oxidized to Fe^3+^ by Cp, secreted into the interstitium through the astrocyte endfeet, and then captured by transferrin that is expressed by cells of the choroid plexus. Iron can also enter the brain through astrocytes (2). A significant amount of Fe^3+^ ions in the CNS circulate attached to low molecular mass molecules secreted by astrocytes such as ascorbate, citrate, or ATP. The CNS also contains a significantly greater amount of NTBI than the periphery. Neurons express high levels of TfRs and acquire the bulk of their iron from transferrin under physiological conditions. Astrocytes, on the other hand, express DMT-1 and internalize Fe^2+^ ions in the form of NTBI. Microglia internalize TBI *via* TfRs as expected, but also utilize the dicarboxylic acid receptor and probably also the lactoferrin receptor. Neurons and other glial cells also acquire NTBI from upregulated DMT-1 under inflammatory conditions (*left part*). Some factors might disrupt this iron balance resulting in iron deficiency (*middle top*) or iron overload (*middle bottom*). The IRP-IRE system regulates iron uptake and storage by modulating the expression of mRNAs coding for iron uptake, storage, and export proteins. When CNS iron levels are low (*right top*), IRP binds to the 3′ IREs of target mRNAs (e.g. TfR1 and DMT1) thus stabilizing the transcript in order to enable translation and the subsequent increase in iron uptake. Concomitant binding to the 5′ IREs of target mRNAs (ferritin, Fpn, ALAS2, HIF-2α, APP, and, possibly, a-synuclein) prevents binding of the 43S preinitiation complex, thus inhibiting translation and reducing iron storage and efflux. In the presence of excess iron in the CNS (*right bottom*), IRP1 incorporates ISCs in order to acquire aconitase activity, while IRP2 is degraded. IRPs thus lose their affinity for IREs, resulting in the degradation of mRNAs with 3′ IRE sequences that code for iron uptake proteins and in the translation of mRNAs with 5′ IREs that code for iron storage and efflux proteins. Figure adapted and get permission from references (Singh et al., 2014; Morris et al., 2018). DMT-1, divalent metal transporter-1; Fpn, ferroportin; Cp, caeruloplasmin; CNS, central nervous system; NTBI, non-transferrin-bound iron; TfR, transferrin receptor; TBI, transferrin-bound iron; LDLR, low density lipoprotein receptor; DCDR, dicarboxylic acid receptor; LAF, lactoferrin; ALAS2, δ-aminolevulinate synthase 2; APP, amyloid precursor protein; HIF-2α, hypoxia-inducible factor-2α; ISC, iron–sulfur cluster; IREs, iron-responsive elements; IRP, iron regulatory protein.

Processes and pathways involved in central nervous system (CNS) iron homeostasis at the cellular level are shown in the left part of Figure 1. After iron enters the brain across the blood–brain barrier and the choroid plexus, the iron is processed by endocytosis (Simpson et al., 2015). Particularly, as a highly specific form of membrane-bound ceruloplasmin, it controls and regulates the activity of ferroportin, which is a ferroxidase enzyme that normally functions as the main copper-carrying protein in the blood and that is also expressed by the adjacent endfeet of astrocytes (McCarthy and Kosman, 2014). Generally, this interaction and regulation between astrocytes and brain capillary epithelial cell hephaestin is enabled by a negative feedback loop (McCarthy and Kosman, 2015). Under physiological conditions, high levels of transferrin receptors (TfRs) are expressed in neurons which could obtain the greater part of their iron from transferrin (Morris et al., 2018). The initial neuronal uptake of transferrin-bound iron (TBI) is achieved by the formation of TfR1 and incoming TBI complex and then is internalized by way of clathrin-mediated endocytosis (Liu et al., 2017). Microglia internalizes TBI by way of TfRs commonly, and by dicarboxylic acid receptor as well as possibly also the lactoferrin receptor. Neurons and other glial cells also obtain non-TBI from upregulated divalent metal transporter-1 (DMT-19) under inflammatory conditions (Morris et al., 2018). At the cellular level (Figure 1, right part), iron metabolism is controlled post-transcriptionally by the IRE (iron responsive element)/IRP (iron regulatory protein) system (Hentze et al., 2010; Wang and Pantopoulos, 2011; Zhou and Tan, 2017). IRP1 and IRP2 are two iron regulatory proteins that bind to IREs in order to regulate the translation or stability of these IRE-containing mRNAs. These mRNAs encode crucial iron metabolic proteins, such as δ-aminolevulinate synthase 2 (ALAS2), H- and L-ferritin, DMT-1, TfR1, ferroportin, hypoxia inducible factor-2α (HIF-2α), and others (Sanchez et al., 2007; Sebastiani and Pantopoulos, 2011; Lane et al., 2015; Yoshinaga et al., 2017). IRPs are activated by ID and other stimuli to bind to cognate IREs, which stabilizes TfR1 and DMT-1 mRNAs and inhibits specific translation of H- and L-ferritin, ferroportin, ALAS2, and HIF-2α mRNAs (Sebastiani and Pantopoulos, 2011).

**Figure 2:**
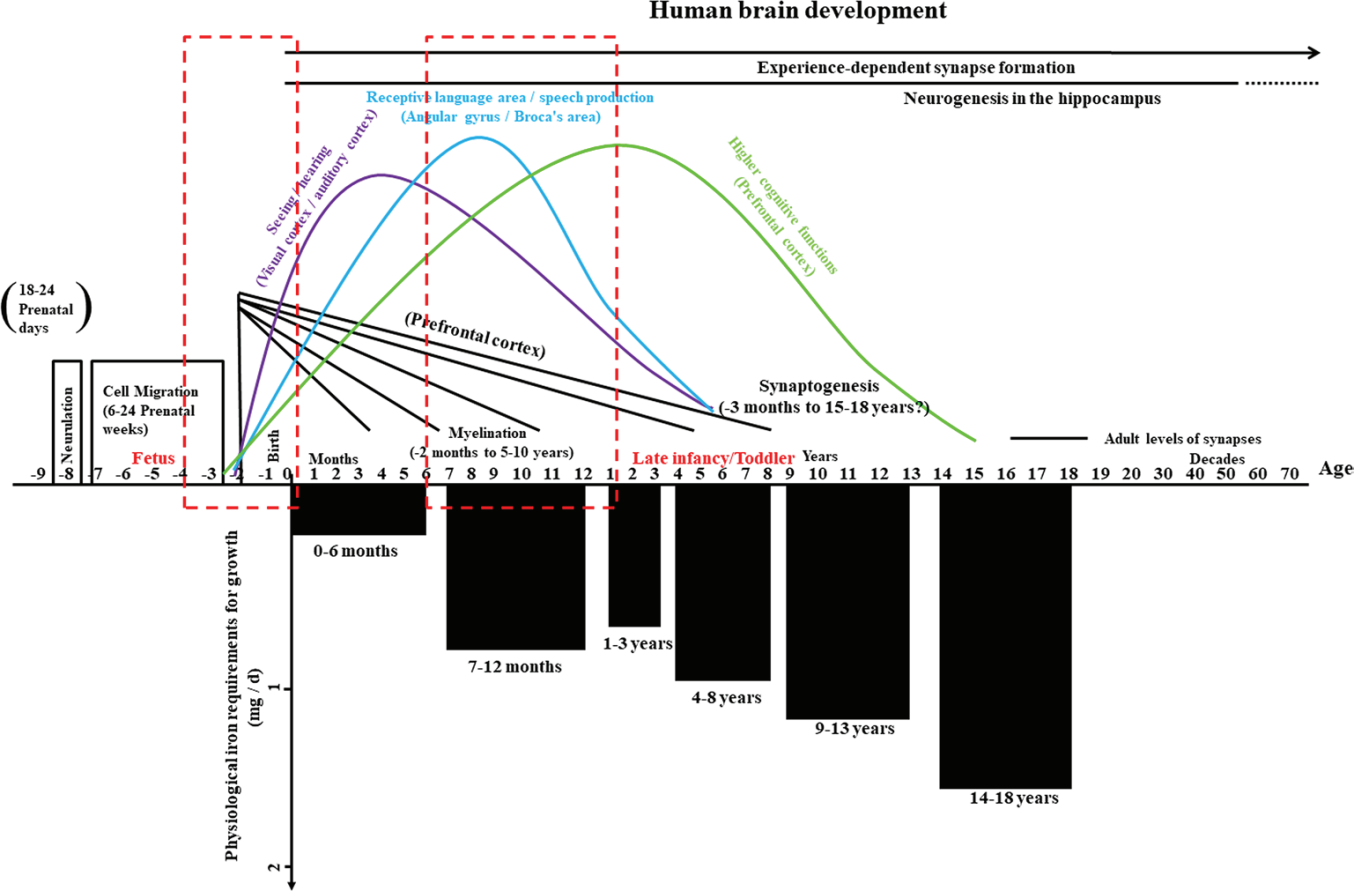
Overview of human brain development and physiological iron requirement for growth in infancy and childhood. The upper part of this graph illustrates the important prenatal events – such as the formation of the neural tube (neurulation) and cell migration, critical aspects (seeing/hearing, receptive language area/speech production, and cognitive functions) of synapse formation and myelination beyond year three, and the formation of synapses based on experience – as well as neurogenesis in a key region of the hippocampus throughout much of life. Periods with high risks for alterations in iron metabolism during early human brain development are highlighted with dashed red boxes. The lower part of the graph shows the physiological iron requirements for growth in different stages during infancy and childhood. Chart adapted and appropriated permission have been obtained from references (Thompson and Nelson, 2001; Georgieff and Innis, 2005; Hider and Kong, 2013).

During the first year of age, the brain experiences an extraordinary transformation from a relatively original into a complex organ. During this period, essential neurodevelopmental processes include synaptogenesis, the organization of neurotransmitter systems, and the onset of myelination, especially within the hippocampus, visual system, and auditory system (Figure 2, top part; Thompson and Nelson, 2001; Georgieff and Innis, 2005), and iron impacts on these developmental processes at multiple levels. Iron is a key nutrient that contributes to fetal and neonatal brain development is associated with critical cellular processes in the immature brain, including the maintenance of neural cell energy status, myelination, and monoamine neurotransmitter homeostasis (Bianco et al., 2008; Todorich et al., 2009; Cheli et al., 2018). The oligodendrocytes are related to myelin production (Sun et al., 2019), and there is an extremely complicated relationship between iron acquisition and myelin production. As a co-factor for cholesterol and lipid biosynthesis, iron directly participates in myelin production and is indirectly involved in oxidative metabolism (which is more likely to occur in oligodendrocytes than in other cells of the brain) (Todorich et al., 2009; Stephenson et al., 2014; Xu et al., 2014).

The neonatal brain is in a highly metabolic state, consuming approximately 60% of the total body oxygen consumption, while the adult brain only consumes about 20% of the total body oxygen consumption (Erecinska and Silver, 1989). Studies in rodents have shown that the high rate of metabolism is iron dependent. After feeding with purified diet of different levels of iron in young rats, cytochrome c and muscle myoglobin show similar degree impact as hemoglobin (Dallman, 1986). During the period of differentiation in neuronal and glial cells, a great amount of metabolic energy is needed for the process of migration, myelination, the establishment of synaptic contacts, and the extension of neuritic processes, especially in rapidly developing brain areas (Simons and Trajkovic, 2006; Li et al., 2011; Zhang et al., 2017a). Iron is mainly located in oligodendrocytes and microglia and is involved in numerous metabolic activities such as myelination, oligodendrocyte maturation, and microglial activation (Zhang et al., 2006; Bishop et al., 2011). Specific iron-requiring enzymes that contribute to maintaining a high level of metabolic activity, including the cytochrome oxidase system, glucose-6-phosphate dehydrogenase, dioxygenase, NADH dehydrogenase, and succinic dehydrogenase, which are all increased in oligodendrocytes compared with other kinds of cells in the brain (Todorich et al., 2009).

Because brain continues to develop during infancy and childhood, diet might have an influence on cognitive ability and behavior (Bryan et al., 2004; Huo et al., 2012; Prado and Dewey, 2014). Iron is one of the most important micronutrients, and meeting its requirement is likely to have an advantageous impact on cognitive development in children (Sachdev et al., 2005). Imbalanced iron status has negative effects on psychological function due to altered activity of iron-containing enzymes in the brain (Benton, 2008; Scott et al., 2018). The neurodevelopmental effects of iron in preterm infants include its influences on the speed of neural processing and on general cognition. For example, early iron supplementation in preterm infants leads to a tendency toward beneficial impact on neurocognitive development at 5.3 years of age; however, as the original study was not designed to assess impacts on neurocognitive development, the power of the study was inadequate to investigate small but possible clinically related improvements, and further research in larger cases to prove this tendency are needed (Steinmacher et al., 2007).

## Impact of Iron Imbalance on Brain Development in Preterm Infants

### Consequences of Iron Deficiency on Brain Function in Preterm Infants

Studies in animal models showed that compromised iron status leads to significant loss of cytochrome c oxidase activity in selected brain structures, especially in the hippocampus and prefrontal area (Carlson et al., 2007; Bastian et al., 2016). In preterm infants, ID has been found to lead to poor physical growth, decreased immunity, and temperature instability (Ohls et al., 2004; Ekiz et al., 2005; Aly et al., 2018). The impacts of ID are extensive and involve numerous organ systems; for example, skeletal muscle dysfunction and altered cardiac contractility might be caused by tissue ID (Stugiewicz et al., 2016; Hoes et al., 2018), but the main concern of early ID is its impact on brain development.

Preterm infants with the lowest quartile cord ferritin concentrations (<76 μg/L) have slower central nerve conduction velocities as measured by auditory brainstem-evoked response (Amin et al., 2010). A clinical trial investigating anemia (Hb ≤ 10 g/dl) and low iron stores (serum ferritin ≤76 μg/L) in preterm infants showed an increased number of abnormal neurologic reflexes (such as glabella reflex, Babinski reflex, plantar grasp, palmar grasp, passive movement of the arms, and passive movement of the legs) at 37 weeks gestational age compared with nonanemic, iron-replete infants (Armony-Sivan et al., 2004). Steinmacher et al. (2007) examined the effects of iron supplementation on neurocognitive development in low birth weight (LBW) premature newborns and found that iron supplementation during the early period (<61 days of age) had a tendency to ameliorate neurocognitive development compared with late supplementation (≥61 days of age). Interestingly, full-term infants with neonatal ID are more likely to be at risk of cognitive deficits, but motor deficits such as fidgety movements (the movements could occur continuously in awake infants except during fussing and crying, and mainly refer to the small amplitude, moderate speed, and variable acceleration that occur in the neck, trunk, and limbs in all directions) seem to predominate in preterm infants (Bruggink et al., 2008).

Recent studies have demonstrated an correlation between ID/iron-deficiency anemia (IDA) and poor neuronal/cognitive consequences in newborns that lasts beyond the period of ID and might affect motor development, recognition memory, social-emotional behavior, and maturation of the CNS (Shafir et al., 2008; Luo et al., 2015; Scott et al., 2018; Otero et al., 2019; Wenger et al., 2019). Studies in term infants and animal models have shown great impacts of perinatal ID on brain development both acutely during the period of deficiency and long-term after iron levels have been restored. These effects include impaired learning and memory, poorer auditory recognition memory, and less cooperative, confident, and persistent personality (Lozoff and Georgieff, 2006; Lozoff et al., 2014; Geng et al., 2015).

More evidence comes from experimental studies that have shown that ID in the time period corresponding to human preterm infants has impacts on cognitive and behavioral function. Perinatal ID (from gestational day 2 until postnatal day 10) in a rat model reduced neuronal metabolic activity and negatively affected memory processing in selected regions of the neonatal brain (Siddappa et al., 2003). Other studies using rodents reported the impact of decreased levels of brain iron on various dopaminergic functions and dopamine-mediated behaviors by measuring brain iron and dopamine transporters and dopamine receptor density, especially when ID occurs in the first 3 weeks after birth (Beard et al., 2003). In addition, Unger et al. (2012) showed that ID in early life (including the gestational period and up to 8 days after birth) leads to acute and persistent changes in regional monoamine concentrations and significant abnormal motor performance in rats. A study using iron-deficient newborn rat pups found that replenishment of iron starting at postnatal day 4 could rectify the influence of ID on both iron levels and monoamine function in a variety of brain regions (Beard et al., 2007). Iron supplementation after the development of hypomyelination, however, is capable of correcting the motor and cognitive abnormalities due to the early ID. These specific deficits could be found in the striatal dopamine system (Youdim, 2008) demonstrating adverse development of the basal ganglia system, which plays critical roles in the initiation and control of movement, as well as in the hippocampus and cortex that are crucial for the functions of memory and cognition (Leisman et al., 2014; Qiu et al., 2016). A study in a rat perinatal ID model found that disrupting dendritic growth in the hippocampus has negative impacts on synaptogenesis (Jorgenson et al., 2003) and that ID also increases the risk of the developing brain even in response to mild hypoxia-ischemia (Rao et al., 2007). A correlation study in weanling rats showed an interesting association between iron, anxiety-like behavior, and dopaminergic system. What is more, nose pokes and rates of habituation were related to prefrontal cortical iron levels, whereas spontaneous activities, which had higher correlation with iron concentrations and the density of dopamine receptors in the ventral midbrain (Han and Kim, 2015). Another study also showed that iron levels in the brain are one of critical factors for anxiety-like behaviors (Breton et al., 2015). Thus, it is clear that ID can be detrimental to brain development and can increase the risk of poor neurodevelopment both in premature newborns and in neonatal animal models. ID might interfere with neurotransmitter metabolism and myelination (Estrada et al., 2014; Deoni et al., 2018), which affects the cognitive and behavioral function of the brain. Moreover, iron plays an important role in the synthesis of hemoglobin and myoglobin, and ID affects the transport and storage of oxygen and energy expenditure, which is adverse to the function of the brain (Wenger et al., 2019). In addition, some of the preclinical works we mentioned above were conducted only in males or females, or they used equal male or female pups in different groups, which indicate that there is no impact of sex differences on iron status in these preclinical studies.

### Consequences of Iron Overload on Brain Development in Preterm Infants

Recent postmortem studies showed that premature neonates who receive multiple blood transfusions often exhibit iron excess (Park and Kim, 2015; Trevino-Baez et al., 2017). Free iron might be released from senescent red blood cells by transfusion hemolysis, and low circulating levels of transferrin and other iron-binding proteins in premature neonates might increase circulation of non-protein-bound iron. It has been shown that there is a tendency for iron excess because of the lacking of the ability of down-regulating iron absorption in neonatal animal models (Leong et al., 2003), and excess iron supplementation in infants might result in higher risk of impaired growth, infection, and disturbed metabolism of other minerals (Domellof, 2007; Stark et al., 2013).

Excess free iron is common in the pathogenesis of intraventricular hemorrhage (IVH) and has been shown to have adverse effects on the brain (Wu et al., 2019). IVH is particularly common in preterm neonates and carries with it high morbidity and mortality (Christian et al., 2016; Song et al., 2016). In the IVH rat model, injection of lysed red blood cells into the ventricles resulted in upregulation of periventricular heme oxygenase-1 (HO-1), while iron injection led to ependymal cell injury with mitochondrial swelling and loss of cilia (Gao et al., 2014). In addition, overexpression of HO-1 is involved in increased activated microglia, which produce more reactive oxygen species (ROS) after hemorrhagic brain injury (Zhang et al., 2017b). Another study with neonatal rats showed that Hb injection led to iron overload in the subventricular zone, which is a site of neuronal stem and progenitor cell proliferation (Strahle et al., 2014). IVH has also been shown to induce substantial damage to the bordering hippocampus in an iron-dependent fashion (Chen et al., 2011), which is likely to be mediated by iron-activated c-Jun N-terminal kinase apoptotic pathways (Garton et al., 2016a,b). In IVH, blood can disperse within the ventricular system, and free iron can accumulate in the ependymal and subependymal regions as indicated by elevated ferritin and iron deposition in these cells (Garton et al., 2016a). Excess free iron released in the red blood cells can also increase the risk of oxidative injury due to hydroxyl radical generation (Lu and Black, 2016; Wu et al., 2019).

Studies have also reported negative effects of iron overload on cognitive development in experimental animal models of preterm infants (Schroder et al., 2013). Kaur et al. (2007) administered iron-fortified formula dose of iron to newborn mice and showed reduced dopamine levels in striatum, neurodegeneration in midbrain and enhanced vulnerability to toxic injury.

Oxidative stress mediated by excessive free iron under conditions of poor antioxidant capacity has been presumed to initiate the progressive loss of brain function in several diseases through the generation of ROS in preterm infants. The possible mechanism behind the negative impact of iron overload is still unknown but might be associated with the pro-oxidative impacts of iron overload or probably an association between iron and other nutrients involved in growth. Iron overload might augment brain oxidative stress status and decrease brain serotonin and dopamine by reacting with hydrogen peroxide and superoxide anions and by producing hydroxyl radicals and ROS as a result of brain cell injury (Elseweidy and Abd El-Baky, 2008; Yu et al., 2011). In these related oxidative stress pathologies, brain cell damage through lipid and protein peroxidation is caused by free iron, which is released from iron stores. Increased levels of lipid and protein peroxidation have been reported in hypoxic neonates, and the more severe the hypoxia the greater the intra-erythrocyte free iron release, ROS production, and oxidative damage (Lu et al., 2015; Sun et al., 2017).

## Risk Factors for Iron Imbalance in Preterm Infants

### Risk Factors for Iron Deficiency in Preterm Infants

#### Shorter Gestation Period

Many factors alone or combined contribute to negative iron balance in preterm infants, which is seen in 25–80% of preterm infants at some point during infancy (Vucic et al., 2013; Ferri et al., 2014). Different from term neonates, in whom the condition typically occurs during the second half of infancy, premature newborns are at risk for developing ID throughout infancy (MacQueen et al., 2017). In normal pregnancy, more than 80% of the iron in the body accumulates during the third trimester of gestation (Widdowson and Spray, 1951), whereas total body iron and hemoglobin content as well as serum and storage iron levels are much lower in premature infants (Siddappa et al., 2007), and premature infants are commonly born with much less than half of term infant’s total body iron at birth. After birth, many preterm infants undergo severe and rapid reduction in hemoglobin (anemia of prematurity) and iron storage due to rapid growth, reduced erythropoiesis, and blood loss due to repeated phlebotomy (Jeon and Sin, 2013). Follow-up studies of premature neonates have indicated that ID can occur within 2 months of discharge from the neonatal intensive care unit (NICU) (Domellof and Georgieff, 2015) because preterm infants begin life in the NICU where a great amount of things can further perturb iron balance. In addition, a clinical study revealed that IDA of prematurity has a significant positive correlation with elevated zinc protoporphyrin/heme ratios (Bjorklund et al., 2017).

#### Timing of Umbilical Cord Clamping at Birth

The amount of blood that is transfused from the placenta to the neonates is very important for the total body iron level. Clinical studies have demonstrated that delayed cord clamping is helpful for establishing iron stores and preventing ID at 3–6 months of age in newborns with normal birth weight (Andersson et al., 2011, 2014; Kc et al., 2017). Delayed cord clamping might be even more crucial in LBW preterm neonates, and Mercer et al. (2006) concluded that delayed cord clamping of premature neonates is involved in decreased demand for blood transfusion, reduced incidence of IVH, and reduced incidence of late-onset sepsis.

#### Maternal Factors

Moderate maternal ID does not affect the iron endowment of their infants, but severe maternal ID does (Lonnerdal et al., 2015), and infants with ID are often born with low iron endowment, indicating the demand for sufficient iron stores at birth. The level of maternal iron status only accounts for about 6% of neonatal iron storage variability at birth, and it is not clear for other reasons that caused highly variation of birth endowment, but prematurity, LBW, intrauterine growth retardation, maternal smoking, and diabetes during pregnancy are likely to be significant factors (Siddappa et al., 2007; Lonnerdal et al., 2015). Neonates born to women with IDA during pregnancy mostly have serum iron concentrations and hematocrits at the same level as neonates born to iron-adequate women, but lower serum ferritin levels are likely to occur in newborns to iron-deficient mothers, indicating lower iron store levels (Shao et al., 2012). However, fetal iron exposure affects early infant growth but does not significantly improve iron status or absorption, and prenatal iron supplementation does not influence iron status of infants at 2 or 5 months of age (Finkelstein et al., 2013), which might indicate that maternal iron status only partly contributes to ID in preterm infants. In addition, the utilization of some drugs during pregnancy could have an impact on neonatal iron metabolism. For example, corticosteroid is used in some pregnant women with certain diseases even though it has a risk of neurodevelopmental impairment in newborns; however, it might increase the iron level (Naigamwalla et al., 2012; Boghossian et al., 2016).

#### Faster Growth

Tissue iron stores are consumed quickly in premature infants demonstrating rapid growth. The inadequate iron stores in these infants can be used up quickly during the first 6–8 weeks after birth, coinciding with the onset of erythropoiesis and rapid catch-up growth (Rao and Georgieff, 2009). The minimum level of hematocrit/Hb ratio is lower and occurs earlier in the majority of preterm infants (gestational age 28–34 weeks) than in those born at a gestational age of 35–42 weeks, while this situation is even worse for premature infants (gestational age 23–28 weeks) due to early net fluid shifts with extravascular fluid moving into the vascular space, leading to dilution and a decrease in the hematocrit/Hb ratio (Jopling et al., 2009). From the age of 20–30 weeks, the average Hb level in premature newborns is lower than in full-term infants at the beginning of this age range, but this difference changes over the next 10 weeks. Interestingly, iron status at birth has no effect on the postnatal growth rate. Ferritin concentrations are initially lower in preterm infants, but these concentrations become similar between preterm infants and term infants over the course of the first year of life (Takala et al., 2010). With the increases in blood volume and Hb mass, the high rate of postnatal catch-up growth needs extra iron supplementation (Rao and Georgieff, 2009).

#### Feeding Mode

The iron concentration in human breast milk is about 0.35 mg/L (Bjorklund et al., 2012). Although iron absorption rate of human breast milk is better than neonate formula, it is obtained only 0.07 mg/kg per day for iron delivery from exclusive breast milk feeding. Although this iron can be well utilized, there is still a potential risk of developing IDA for neonates who are breastfed for more than 4–6 months without receiving iron-fortified complementary foods or iron supplements (Lonnerdal et al., 2015). Most premature neonates are not solely breastfed longer than 3 months and thus are dependent on iron in preterm and post-discharge formula. Low iron formulas containing less than 5 mg/L of iron do not satisfy the iron demands of the growing premature neonate (Baker et al., 2010). Newborns with poor iron status might require more iron; however, it is not clear if higher levels of iron fortification formula will lead to increased iron levels in infants who fed with formula up to 6 months old (Domellof et al., 2001). Interestingly, a recent study reported that preterm neonates have adequate iron storage at birth and at 2 months old and that they are not likely to require iron supplementation until at least 2 months of age (Saha et al., 2016). As mentioned above, many preterm infant characteristics and/or maternal factors have negative effects on iron status (Figure 1, middle upper). Although ID is more common in preterm neonates, other factors that cause iron overload should be considered.

### Risk Factors for Iron Overload in Preterm Infants

Although IDA has been considered to be an issue in growing premature neonates, the impact of iron overload has not been thoroughly investigated. As an invasive test, liver biopsy is the gold standard for diagnosing iron excess, while serum ferritin level, which is a helpful biochemical assay, is usually utilized as a surrogate indicator to assess and guide treatment of iron excess in older children (Fleming and Ponka, 2012).

#### Medicinal Erythrocyte Transfusion

This is the main factor resulting in iron overload in preterm infants. Premature neonates that receive more erythrocyte transfusions not only could replace phlebotomy losses but also maintain certain level of Hb concentrations. Physicians use erythrocyte transfusions as a frequent intervention when treating preterm infants with very low birth weight (birth weight <1,500 g) (Trevino-Baez et al., 2017). This poses several risks, including iron overload (Park and Kim, 2015), because excess iron is not able to be eliminated by physiological pathways, even though the iron released after degradation of the transfused red blood cells increases body iron storage. Indeed, serum ferritin levels increase significantly with the first month after birth in premature neonates who receive multiple red blood cell transfusions, and there is a greater risk of iron excess in exposed preterm neonates in comparison with nonexposed infants (Herzlich et al., 2016).

#### Inappropriate Infant Formulas

The majority of neonate formulas contain 4–12 mg of iron/L, which is 10–60 times more compared with the concentration of iron in human breast milk. It might be debated whether neonate formula should contain such an excess of iron during the period of the first 6 months, which has no beneficial effect, so as to fit perceived iron demands at 6–12 months of life (Lonnerdal et al., 2015). A clinical study demonstrated that newborns with lower Hb (<106 g/L) benefit from neonate formula with a higher concentration of iron and indicated superior developmental outcomes at 10 years of age compared with those infants who were fed with formula with less iron concentration from 6 to 12 months of life (Lozoff et al., 2012). However, the infants with an initial Hb higher than 128 g/L had worse scores (especially for spatial memory and visual-motor integration) when formula with higher levels of iron was given.

#### Sex

Significant sex differences in iron overload have been observed during infancy. Molloy et al. studied 60 growing, stable premature neonates who had increased iron indices and found significantly greater increases in male infants (Molloy et al., 2009). Ziegler et al. also reported differences in iron levels between males and females and found sex differences in mean corpuscular volume (Ziegler et al., 2014).

In general, iatrogenic factors are responsible for excess iron accumulation in premature neonates, but other risk factors such as medical iron supplementation and infant formula containing a higher level of iron should not be ignored (Figure 1, middle bottom).

## Indicators for Iron Status in Clinical Practice

Screening the iron status of mothers, neonates, and children is necessary to avoid long-term adverse health impacts for mothers and children, especially neurodevelopment abnormalities in the child caused by ID. In view of the current lack of sufficient evidence, the standard for describing iron status clinically for healthy preterm infants is still unknown. Even though the iron level marker in amniotic fluid might be a potential indicator during pregnancy, some factors such as the expression of fetal oxidative stress factors might also significantly affect this trend Gazzolo et al. (2005). As summarized above, iron imbalance including both deficiency and overload has severe impacts on brain development, and thus it seems essential to establish the association between potential indicators such as non-TBI and neurological outcomes in infants.

Hb and ferritin are used as indicators of iron status in infants. Because most physiological changes in iron status and erythrocyte morphology occur during early development, age-specific cutoffs indicators of iron status should be utilized for preterm neonates with LBW (Table 1). In these clinical indicators for iron status, we could see that the thresholds appear to decrease slightly with advancing postnatal age, with the exception of the 2-month value (for example, Hb). For preterm infants with LBW, multiple factors can result in ID and iron overload even if the infants are without pathological disease, and so these clinical indicators are a little lower due to the different iron status in infants who choose to enroll in these studies (Domellof et al., 2002; Siddappa et al., 2007).

**Table 1:** Clinical indicators for iron imbalance in LBW preterm infants at different ages.

	Newborn	2 months	4 months	6–24 months
ID: SF (μg/L)	<35	<40	<20	<10–12
IDA: Hb (g/L)	<135	<90	<105	<105
Iron overload: SF (μg/L)	>300	>300	>250	>200

ID, iron deficiency; LBW, low birth weight; SF, serum ferritin; IDA, iron deficiency anemia; Hb, hemoglobin. Data adapted from Domellof et al. (2002) and Siddappa et al. (2007).

However, because ID is highly prevalent throughout the world, the indicators for detecting iron status are initially focused on identifying whether ID occurs. The present clinical indicators and proposed tests for monitoring ID include hematologic and non-hematologic measurements (Table 2). These indicators are changing gradually before individuals become more iron-deficient. However, to protect the developing brain, the two main points in this process are worthy to be noted. As summarized by Georgieff (2017), first, none of the markers directly index iron levels in brain tissue. In addition, it is unclear whether the brain is lacking iron in this process from sufficient to anemia unless it occurs prior to obvious anemia. Brain iron status detected by direct imaging would be expensive, as well as not currently possible due to the low sensitivity of MRI technology, which is not able to find low iron levels, although it can reveal iron excess (Langkammer et al., 2010). Neurobehavioral tests are attractive candidates as bio-indicators of brain iron status because it reflects iron-specific brain functions (Georgieff, 2017). However, an imbalance in other nutrients such as copper, zinc, and iodine can also lead to similar abnormal neurobehavior (Hagmeyer et al., 2014; Ganaie et al., 2015; Petro et al., 2016; Iglesias et al., 2018). Thus, none of the proper neurobehavioral tests can be used as a direct iron-specific indicator for indexing brain functions. However, some neurobehavioral tests such as the hesitancy and anxiety-like behavior tests might reflect iron status by affecting dopamine receptor/transporter status in animal models (Beard et al., 2002).

**Table 2:** Clinical indicators for monitoring iron deficiency and iron deficiency anemia.

Condition	Physiology	Current test	Proposed test
Mild ID	Mobilized available iron	↓Serum iron, ↓SF	↓Hepcidin, ↓CHr, Perl’s staining (−)
Moderate ID	Increased iron delivery	↑TIBC, ↓TSAT, ↑sTfR	↓Hepcidin, ↓CHr, Perl’s staining (−)
Moderate to severe ID	Altered RBC morphology	↓MCV, ↑ZPP	↓Hepcidin, ↓CHr, Perl’s staining (−)
IDA	Impaired RBC production	↓Hb	↓↓Hepcidin, ↓CHr, Perl’s staining (−)

ID, iron deficiency; IDA, iron deficiency anemia; MCV, mean corpuscular volume; RBC, red blood cell; SF, serum ferritin; sTfR, soluble transferrin receptor; TIBC, total iron binding capacity; TSAT, transferrin saturation; ZPP, zinc protoporphyrin; Hb, hemoglobin; CHr, reticulocyte hemoglobin concentration; Perl’s staining, Perl’s staining of bone marrow for iron. ↑, increased; ↓, reduced; (−), negative. Data adapted from Baker et al. (2010), Wilson and Sloan (2015), and Georgieff (2017).

## Current Recommendations for Iron Supplementation in Preterm Infants

The physiological iron requirements for growth vary in different stages during infancy and childhood (Hider and Kong, 2013; Figure 2, bottom part). It is assumed that the iron absorption rate can be up to 50% from human breast milk and that it is about 10% from neonate formula and iron-fortified complementary foods (Saarinen et al., 1977), but some factors, as summarized in this article, probably result in iron imbalance. Thus, proper iron supplementation is crucial, especially for preterm infants who are at high risk of iron imbalance. The recommendation for iron supplementation in preterm infants from the American Academy of Pediatrics is that breastfed premature infant requires to be supplemented with 2 mg/kg of iron per day from 1 to 12 months old. Premature neonates will get about 1.8–2.2 mg/kg/day of iron by feeding a standard neonate formula (14.6 mg/L of iron) or a standard full-term neonate formula (12.0 mg/L of iron; Baker et al., 2010).

Despite the use of iron-containing formulas, some preterm infants develop ID during the first year of life. Thus, some formula-fed premature neonates might require an extra iron supplement; nevertheless, there is insufficient evidence to confirm this as a common recommendation at this time. In clinical practice, premature neonates who receive multiple blood transfusions are exceptions, so they might not require any iron supplementation (Baker et al., 2010).

Another recommendation for iron supplementation in preterm infants from the European Society for Pediatric Gastroenterology, Hepatology, and Nutrition (ESPGHAN) concluded that iron supplementation of preterm infants with slight LBW at a dose of 1–2 mg/kg/day up to 6 months has few adverse effects and decreases the risk for later adverse cognitive and behavioral performance (Lozoff et al., 2006; Domellof et al., 2014). According to the requirement of ESPGHAN enteral nutrition guidelines for premature neonates, newborn with birth weight <2,000 g should be supplemented with 2–3 mg/kg of iron (Agostoni et al., 2010). Because iron stores are usually used up at about 6 months old, iron-rich complementary foods are recommended. Even if iron-fortified follow-on formulas should be supplemented, determining the optimal level of iron in follow-on formulas still lacks sufficient evidence. When the infants grow up to 6 months old, it is necessary to give them iron-rich food. Before 12 months of age, unmodified cow’s milk is not suggested to infants as the main milk drink (Agostoni et al., 2010).

## Remarks

Preterm infants are at high risk of iron imbalance, and ID and iron overload are important nutritional issues in preterm infants. The potential risk for neurodevelopmental abnormalities caused by ID requires regular screening and preventive measures. It is also beneficial and safe for preterm infants to be given iron supplementation. On the other side, iron overload is another significant concern in preterm infants; however, the management of premature infants who have excess iron has not been well investigated. Because iron levels in premature neonates vary greatly, we should monitor its status carefully during neonatal and post-discharge periods. The gestational age-specific clinical indicators for evaluating iron status and the neurobehavioral examinations reflecting iron-specific brain function are necessary to be developed. In the previous iron status and iron supplementation studies, most of them were conducted before the period of increased survival of high-risk premature neonates. Thus, randomized and well-controlled trials are required to establish iron supplement guidelines for these preterm infants.

## Author Contributions

CZ devised the review. YWa, YWu, and TL reviewed the literature and wrote the manuscript drafts. XW and CZ substantially contributed to the literature review and the writing of this manuscript.

### Conflict of Interest Statement

The authors declare that the research was conducted in the absence of any commercial or financial relationships that could be construed as a potential conflict of interest.
